# Patterns of circulating cytokines after 2 types of exercise in active type-1 diabetic patients

**DOI:** 10.1007/s13105-025-01123-5

**Published:** 2025-10-10

**Authors:** María Herranz-López, Rodrigo Martín-San Agustín, Mariló Olivares-Vicente, Laura Pardines-Oltra, Alba Cuerda Del Pino, Jorge Bondia, Paolo Rossetti, F. Javier Ampudia-Blasco, Enrique Roche

**Affiliations:** 1 Clinimetry and Technological Development in Therapeutic Exercise Research Group (CLIDET), Department of Physiotherapy, University of Valencia, Valencia, 46010 Spain; 2 Instituto de Investigación, Desarrollo e Innovación en Biotecnología Sanitaria de Elche (IDiBE), Miguel Hernández University (UMH), Elche, 03202 Spain; 3 Centro de Investigación Biomédica en Red de Diabetes y Enfermedades Metabólicas Asociadas (CIBERDEM), CB17/08/00004, Instituto de Salud Carlos III, Madrid, 41092 Spain; 4 Instituto Universitario de Automática e Informática Industrial, Universitat Politècnica de Valencia, Valencia, 46022 Spain; 5 Department of Endocrinology & Nutrition, University and Polytechnic La Fe Hospital of Valencia, Valencia, 46026 Spain; 6 INCLIVA Biomedical Research Institute, Valencia, 46010 Spain; 7 Department of Medicine, University of Valencia, Valencia, 46010 Spain; 8 Research Group “Nutrition and Physical Activity”, Spanish Nutrition Society “SEÑ”, Madrid, 28010 Spain; 9 Department of Applied Biology-Nutrition, Institute of Bioengineering, Miguel Hernandez University, Elche, 03202 Spain; 10 Alicante Institute for Health and Biomedical Research (ISABIAL), Alicante, 03010 Spain; 11 CIBER Fisiopatología de la Obesidad y Nutrición (CIBEROBN), Instituto de Salud Carlos III (ISCIII), Madrid, 28029 Spain

**Keywords:** Aerobic exercise, High intensity interval training, Inflammatory cytokines, Physical activity, Protein carbonyls

## Abstract

Aerobic training (beneficial for type 1 diabetes mellitus (T1DM) patients) could result boring, leading to inactivity or suboptimal performance. Therefore, High-Intensity Interval Training (HIIT) could be an appealing alternative. The present study aimed to compare modulation of certain inflammatory cytokines in T1DM participants performing aerobic vs. HIIT routines. We recruited 26 T1DM male subjects: ages 18–40, T1DM *≥* 2 years, glycated hemoglobin < 8.5%, stable insulin regimen for 6 months, minimum 90 min weekly physical activity and completion of International Physical Activity Questionnaire (IPAQ). IPAQ:2 corresponds to moderate activity and IPAQ:3 to intense physical activity. Participants performed a single aerobic or HIIT session separated by at least 72 h. Blood plasma samples were collected 20 min before and after each session. Cytokines were measured using LUMINEX technique. After aerobic exercise, pro-inflammatory cytokines interleukin (IL)-1β, IL-6, IL-7, IL-8, IL-17 A, tumor necrosis factor-α (TNF-α) and interferon-γ (IFN-γ) showed no significant differences in participants (IPAQ:2 and 3). Only anti-inflammatory cytokine IL-2 increased significantly in IPAQ:3 participants. Compared to pre-exercise, post-exercise HIIT situation presented a similar pattern. The 4 participants (IPAQ:3) that voluntarily followed a 12-week HIIT routine, showed significant increases in IL-7, IL-8, and TNF- α, and the detection of the anti-inflammatory cytokine IL-22. Altogether, these results suggest that HIIT favors the presence of some pro-inflammatory cytokines. The anti-inflammatory action of certain cytokines, such as IL-22, should be considered for a possible compensatory action. Nevertheless, programs of interval exercises at moderate intensity could be at the moment a safe option for T1DM patients.

## Key Points

- Significant increase was observed only for IL-2 after aerobic exercise in participants of IPAQ:3 level.
- Pro-inflammatory cytokine levels do not change significantly after aerobic or HIIT routines, except for IL-6, IL-7 and IL-8 at long-term in participants of IPAQ:3 level.
- Anti-inflammatory cytokines IL-2 (short-term) and IL-22 (long-term) show significant changes after aerobic and HIIT exercises in participants of IPAQ:3 level.

## Introduction

The autoimmune destruction of β-cells in the endocrine pancreas marks the onset of type 1 diabetes mellitus (T1DM). This stage is characterized by inflammation of the islets (insulitis), increased expression of human leukocyte antigen (HLA), and the presence of β-cell autoantigens [1–3]. CD4 + and CD8 + T-lymphocytes, macrophages and components of the complement system infiltrate into islet structure [4]. Elevated levels of HLA and antigen transporter proteins are associated with enhanced activity in presenting autoantigens, which is relevant in the context of the autoimmune attack. Altogether, this autoimmune process culminates in the total destruction of the β-cells and absence of insulin. T lymphocytes are responsible for controlling the processes of self-tolerance. However, in T1DM, this control fails, initiating an inflammatory cascade (insulitis) and progressing to islet destruction. The pathogenic immune response is mediated by Th1 lymphocytes (a subpopulation of CD4+), while protection is due to Th2 lymphocytes. In T1DM, the balance shifts in favor of Th1 lymphocytes, which infiltrate islet tissue [5]. Th1 lymphocytes secrete pro-inflammatory cytokines, meanwhile anti-inflammatory cytokines are produced by Th2 lymphocytes [6].

Therefore, a pro-inflammatory state predominates in the progression of the disease, as in addition to local inflammation in the pancreas, people with T1DM may experience chronic low-grade systemic inflammation. This condition can contribute to long-term complications in various organs and systems such as kidneys, eyes, as well as nervous and cardiovascular systems [7, 8]. In this context, endothelial cells of blood vessels, as well as Schwann cells and renal mesangial cells, are unable to limit glucose uptake, making them more vulnerable to the effects of persistent hyperglycemia. These toxic effects are exacerbated by the formation of reactive oxygen species (ROS) from both mitochondrial and non-mitochondrial sources, which cannot be mitigated due to the low activity of antioxidant defenses [9]. Consequently, hyperglycemia accelerates the oxidative imbalance that occurs in T1DM through various mechanisms, such as activation of the hexosamine and polyol pathways and uncontrolled glycation end products [10]. Additionally, products of the lipoxygenase pathway are involved in the pathogenesis of T1DM, mediating the action of pro-inflammatory cytokines [11].

Exercise can alleviate both inflammation and oxidative stress damage in T1DM patients [12]. Acute exercise produces a short-term inflammatory response that is accompanied by increases in interleukin (IL)−6 and tumor necrosis factor-α (TNF-α), as well as ROS. However, at long-term (regular exercise) this pro-inflammatory response is followed by an anti-inflammatory effect with increases in anti-inflammatory cytokines, such as IL-4 and IL-10 [13]. In addition, exercise can activate antioxidant defenses through a hormesis mechanism. Thus, the free radicals formed during physical activity would be beneficial, as they would activate the genes that encode antioxidant enzymes. This activation occurs via the nuclear factor erythroid 2-related factor (Nrf2), which is trapped in the cytosol by the repressor Kelch-like ECH-associated protein 1 (Keap1). The free radicals formed during exercise would release Nrf2, allowing it to migrate to the nucleus and activate the genes encoding endogenous antioxidants [14].

Therefore, controlling blood glucose levels as well as the inflammatory and prooxidative state is essential to slow the progression of the disease. Exogenous insulin injections can help control blood glucose levels, although this control is not as precise as that achieved by endogenous insulin release. Therefore, complementary mechanisms might cooperate to control glycemia. In this context, it is well known that physical activity can modulate blood glucose levels by acquisition of long-term physiological adaptations that allow better control of blood glucose and increased cellular sensitivity to the action of insulin [15, 16]. At the onset of exercise, myocytes use their intracellular reserves of ATP, phosphocreatine, and glycogen as consecutive energy sources. Alternatively, skeletal muscle also obtains energy from plasma glucose [17]. In addition, the contraction performed by skeletal muscle during exercise promotes the incorporation of vesicles containing the glucose transporter GLUT4 into the membrane [18], allowing for better glucose uptake. Altogether, this is instrumental in glycemia control in T1DM that consequently helps to diminish inflammation and oxidative stress.

For all these reasons, the debate focuses on determining the most suitable type of exercise for people with T1DM, as well as its duration and intensity. In this regard, aerobic exercises are well-accepted by people with T1DM. During an aerobic routine (i.e. 120 min, 60% V̇O_2_max), the muscle primarily metabolizes glucose in hyperglycemia, compared to normoglycemic situations where the muscle metabolizes both carbohydrates and lipids [19]. Since aerobic training is capable of modulating hyperglycemia, this ultimately should result in a reduction of glycated hemoglobin (HbA1c) levels, although this is still a matter of debate [20]. Altogether, this also leads to a decrease in the doses of injected insulin following aerobic exercise compared to the initial situation [21]. Although people with T1DM have higher levels of oxidative stress than healthy people, performing aerobic exercise does not seem to worsen this condition, at least in the short term [22]. Therefore, aerobic exercise appears to enhance antioxidant defense and reduce fatigue in people with T1DM. Additionally, it promotes improvements in cardiovascular parameters such as VO_2_max, maximum heart rate, ejection fraction, and cardiac output [23].

However, aerobic exercise may be perceived as monotonous and boring for some people with T1DM, reducing their motivation. Therefore, alternative exercises such as High-Intensity Interval Training (HIIT) might be appealing to certain patients. However, fewer studies have been conducted on the role of HIIT in controlling physiological parameters in patients with T1DM compared to studies on endurance training. In this regard, we conducted a pilot study with continuous glucose monitoring in subjects performing a HIIT routine using elastic resistance bands for 3 months, observing improvement of values related to glycemic control [24]. In the current study, we have aimed to expand our research investigating how the levels of certain inflammatory cytokines are modulated in active T1DM subjects performing an aerobic exercise compared to same subjects performing HIIT.

## Materials and methods

### Experimental design

Twenty-six T1DM male subjects participated in the study (Table 1) were recruited from 70 volunteers. More details of the selection process (CONSORT and flow diagram) are indicated in a complementary study focused in the continuous glucose monitoring followed by participants [25]. The inclusion criteria were: males aged between 18 and 40 years, with T1DM for more than 2 years, HbA1c less than 8.5%, stable insulin regimen in the last 6 months with less than 20% change in the total daily insulin dose, multiple daily injections, and weekly physical activity of 90 min or more, but without practicing any sports as an amateur or professional. Excluded participants were those whose clinical conditions or medication (other than insulin) affected glycemic control. All participants were informed about the potential risks and benefits of the project and signed an informed consent form. The experimental protocol was approved by the Ethics Committee of the University of Valencia (Spain) (Ref. 1587001).

**Table 1:** Circulating and physical activity parameters of subjects participating in the study (*n* = 26)

Parameter (units)	
Age (years)	29.3 *±* 6.3
Diabetes duration (years)	14.8 *±* 6.1
Weight (Kg)	80,80 *±* 13.38
Height (m)	1.80 *±* 0.06
BMI (Kg/m ^2^)	25.08 *±* 3.53
HbA1c (%)	7.19 *±* 1.03
Daily insulin administered (IU)	52.69 *±* 16.00
Daily insulin administered (IU/weight Kg)	0.66 *±* 0.20
IPAQ score (number of subjects)	2 (*n* = 12) 3 (*n* = 14)
Cholesterol (mg/dL)	164.15 *±* 25.34
Triglycerides (mg/dL)	84.62 *±* 59.79
HDL (mg/dL)	52.58 *±* 10.81
LDL (mg/dL)	105.54 *±* 20.36
Cortisol (µg/dL)	16.17 *±* 4.31

At the time of their recruitment, participants completed an IPAQ (International Physical Activity Questionnaire) which measures the degree of physical activity they usually perform. According to this questionnaire, the degree of physical activity can be divided into 3 levels: level 1 corresponds to a low level of physical activity, level 2 to moderate physical activity, and level 3 to intense physical activity. Participants in this within-group study with repeated measures were in levels 2 and 3 (Table 1). Then participants performed a single HIIT or aerobic session in a randomized order, separated by at least 72 h to avoid interactions. The blood plasma samples were taken at the Clinical Research Laboratory of the Physiotherapy Department of the University of Valencia (Spain), 20 min before and after each exercise routine. Each extraction was performed in a different arm.

Prior to training sessions, the participants performed a graded exercise test to determine the power output on the cycle-ergometer for the session until voluntary exhaustion [26]. The aerobic routine began with a 3-min rest period (0 Watts) on the cycle-ergometer, followed by a 3-min warm-up at 60 Watts, and a stepwise increase in intensity of 20 Watts/min until reaching the power determined in the graded test as the aerobic threshold [26]. This target workload was maintained for 30 min. For the HIIT exercise, participants performed a 3-min warm-up (60 Watts) on the cycle-ergometer and 15 shoulder flexion-extensions without load. The session consisted of two series of elastic band exercises at 20-s intervals separated by 10 s of rest, with a 3-min rest between the two series (i.e., 4 min in total) with four upper limb exercises (bench press, seated dumbbell press, shoulder press and seated rowing) and four lower limb exercises (squats, stiff-legged deadlift, hamstring curl and quadriceps curl) [24]. The length and color of the elastic band, which defines its resistance, was chosen by the participants to ensure a maximum effort during the 20 s of each exercise. The protocol was a modification of the HIIT protocols used by previous authors, replacing weight exercises with TheraBand CLX^®^ exercises [24]. Participants were encouraged to perform as many repetitions at full speed per interval as possible while maintaining correct form. For the rating of perceived exertion (RPE), the Thera-Band resistance exercise scale of perceived exertion with Thera-Band resistance bands was used [27] with the intensity set for HIIT RPE *≥* 8.

After the training sessions, participants were invited to participate in a long-term HIIT routine, previously tested in [24]. Only 4 participants agreed to perform this routine for 12 weeks. All exercise sessions were conducted at the Clinical Research Laboratory of the Department of Physiotherapy at the University of Valencia, Spain, and were supervised by a physiotherapist trained in this type of exercise methodology. Briefly, the long-term HIIT routine consisted of 3 weekly HIIT sessions using elastic bands, identical to those described above, with 48-h intervals between sessions over 12 weeks. The routine included one series of 4 min of selected exercises during the first 4 weeks, 2 series in the following 4 weeks, and 3 series in the final 4 weeks. A fasting blood sample was obtained at the end of this 12-week period.

Blood glucose levels were monitored during each session, at the beginning and at the end, and 60 min after (recovery period). If glucose levels were less than 60 mg/dL at any time, the exercise session was either not started or was stopped to prevent hypoglycemia. In case of mild hypoglycemia (60–69 mg/dL), participants drunk 200 mL of orange juice containing 10.4 g/100 mL of carbohydrates, and blood glucose was checked again after 10 min. If glucose levels were not above 70 mg/dL, additional 100 mL of juice were administered, and glycemia was checked again after 10 min. All participants displayed no big fluctuations in glycemia, confirming previous observations [24]. Once the plasma samples were obtained, different cytokines were determined as indicated below.

## Plasma determinations: cytokines and protein carbonyls

Primary outcome measures include a panel of cytokines related to inflammatory processes mainly occurring in T1DM. The high sensitivity immunoassay kit ProcartaPlex^®^ Mix&Match-9 from eBioscience division of Affymetrix was used to determine the levels of IL-1β, IL-2, IL-4, IL-6, IL-7, IL-8, IL-10, IL-17 A, IL-22, TNF-α and interferon-γ (IFN-γ). To measure the levels of the indicated cytokines, the Luminex MAGPIX system (Thermo Fisher Scientific) was used [28].

Protein carbonyl derivatives were measured in plasma using the Protein Carbonyl Content Assay Kit (MAK094, Sigma-Aldrich). Determination is based in the derivatization of the protein carbonyl groups with 2,4-dinitrophenylhydrazine until the stable formation of dinitrophenyl hydrazone adducts, which can be detected spectrophotometrically at 375 nm [29].

### Statistical analysis

Normal distribution of variables was determined according to Shapiro-Wilk and Kolmogorov-Smirnov tests. Levene test was used for homogeneity analysis. Since cytokines and protein carbonyls do not follow a normal distribution, intra-group differences were determined through Kruskal-Wallis non-parametric test and multiple comparison probe of Dunnett, and results were expressed as median (interquartile range). Statistical significant differences were considered for *p* < 0.05. The software GraphPad Prism 8 (GraphPad, San Diego, CA) was used for data analysis.

## Results

Different patterns of circulating cytokines were observed for the different types of exercise in T1DM participants. IL-1β, IL-4, IL-6, IL-7, IL-8, IL-17 A, TNF-α and IFN-γ displayed no significant differences after the aerobic exercise, compared to the pre-exercise situation (Table 2). In this exercise test, only IL-2 presented significant increases post-exercise in IPAQ:3 participants (Table 2). Regarding HIIT exercise, a similar pattern was observed (Table 3). Regarding protein carbonyls, no significant changes were observed after both exercises (Tables 2 and 3). After this general presentation, a more detailed analysis of the obtained results will be conducted.

**Table 2:** Cytokine and protein carbonyl levels before (PRE) and after (POST) aerobic exercise (AERO) for the groups according to their habitual physical activity levels determined through IPAQ score (2: moderate, 3: intense)

Cytokines (pg/mL)	PRE-AERO (IPAQ:2) (*n* = 12)	POST-AERO (IPAQ:2) (*n* = 12)	*p*	PRE-AERO (IPAQ:3) (*n* = 14)	POST-AERO (IPAQ:3) (*n* = 14)	*p*
Pro-inflammatory						
IL-1β	0.05 (0-0.09)	ND		2.36 (1.35–17.51)	0.43 (0.22–1.60)	> 0.99
IL-17 A	0.29 (0-1.51)	0.58 (0.29–1.86)	> 0.99	2.11 (0.46–11.83)	0.80 (0.17–3.25)	> 0.99
TNF-α	ND	0.01 (0-0.01)	> 0.99	0.01 (0-0.20)	0.64 (0.03–0.95)	> 0.99
IFN-γ	1.23 (0-2.42)	ND		5.21 (2.03–7.30)	0.61 (0.31–2.37)	> 0.99
Anti-inflammatory						
IL-2	0.12 (0-0.18)	4.12 (2.06–6.19)	> 0.99	2.33 (0.76–3.76)	5.67* (5.60-10.25)	< 0.05
IL-4	0.35 (0-1.03)	1.37 (0.68–4.30)	> 0.99	1.21 (1.00-1.72)	1.76 (0.73–4.83)	> 0.99
IL-10	0.03 (0-0.17)	0.10 (0.05–0.15)	> 0.99	0.28 (0.07–0.51)	0.22 (0.09–0.50)	> 0.99
IL-22	ND	ND		ND	ND	
Homeostatic						
IL-6	7.75 (3.11–11.98)	6.88 (4.33–7.98)	> 0.99	10.64 (2.01–14.11)	2.93 (0.75–11.90)	< 0.73
IL-7	0.59 (0.28–0.64)	1.92 (0.36–2.94)	> 0.99	0.41 (0.24–0.62)	1.19 (0.55–2.07)	< 0.62
IL-8	0.47 (0.11–0.85)	1.16 (0.80–2.10)	< 0.58	0.86 (0.32–1.05)	1.17 (0.82–1.97)	< 0.53
Protein carbonyls (nmols/mg protein)						
	9.01 (6.45–11.67)	7.42 (5.37–9.92)	> 0.99	9.64 (5.65–11.23)	8.47 (6.10-10.95)	> 0.99

Data are expressed as median (interquartil range). ND, no detected. (*) Significant (*p* < 0.05) respect to PRE-AERO (IPAQ:3)

**Table 3:** Cytokine and protein carbonyl levels before (PRE) and after (POST) HIIT exercise for the groups according to their habitual physical activity levels determined through IPAQ score (2: moderate, 3: intense)

Cytokines (pg/mL)	PRE-HIIT (IPAQ:2) (*n* = 12)	POST-HIIT (IPAQ:2) (*n* = 12)	*p*	PRE-HIIT (IPAQ:3) (*n* = 14)	POST-HIIT (IPAQ:3) (*n* = 14)	*p*	LT-HIIT (*n* = 4)	*p*
Pro-inflammatory								
IL-1β	0.15 (0.09–0.23)	1.00 (0.50–1.20)	> 0.99	0.19 (0.09–0.28)	1.60 (0.13–2.66)	< 0.33	0.55 (0.36–0.85)	< 0.65
IL-17 A	0.46 (0.24–0.46)	1.39 (0.69–2.08)	> 0.99	0.41 (0.19–0.47)	0.88 (0.51–1.39)	> 0.99	0.66 (0.35–1.13)	> 0.99
TNF-α	0.16 (0.07–0.18)	0.21 (0.11–0.55)	> 0.99	0.22 (0.05–0.28)	0.64 (0.21–1.25)	< 0.67	0.53* (0.40–1.20)	< 0.05
IFN-γ	3.22 (1.61–4.72)	ND		1.27 (0.61–2.02)	0.31 (0.31–1.34)	< 0.89	0.11 (0.05–0.16)	< 0.89
Anti-inflammatory								
IL-2	0.37 (0.18–0.81)	ND		0.39 (0.01–1.43)	11.57* (8.93–14.92)	< 0.05	1.51 (0.82–2.85)	< 0.16
IL-4	0.24 (0.01–0.33)	3.73 (1.86–5.59)	> 0.99	0.21 (0.01–0.38)	1.25 (0.36–1.95)	< 0.89	1.44 (0.72–2.16)	> 0.99
IL-10	0.03 (0.06–0.99)	0.09 (0.05–0.14)	< 0.89	0.22 (0.07–0.51)	0.15 (0.11–1.17)	> 0.99	0.71 (0.62–0.74)	< 0.96
IL-22	ND	ND		ND	ND		2.49 (0.29–5.08)	
Homeostatic								
IL-6	4.88 (3.26–8.52)	5.91 (4.50–9.39)	> 0.99	6.54 (2.01–9.85)	2.10 (1.42–2.69)	< 0.96	2.47 (1.65–6.16)	< 0.84
IL-7	0.17 (0.07–0.23)	0.18 (0.15–0.85)	< 0.89	0.21 (0.04–0.26)	1.25 (0.58–1.71)	< 0.61	8.00* (6.42–9.75)	< 0.05
IL-8	1.52 (1.12–1.68)	2.04 (1.72–2.08)	> 0.99	0.96 (0.90–1.65)	2.16 (1.45–3.30)	< 0.52	13.01* (10.76–13.24)	< 0.05
Protein carbonyls (nmol/mg protein)								
	21.60 (6.08–22.05)	13.20 (8.28–16.89)	> 0.99	10.46 (2.97–15.99)	4.92 (2.94–5.29)	< 0.61	7.42 (3.25–8.07)	> 0.99

Data are expressed as median (interquartil range). ND, no detected. (*) Significant (*p* < 0.05) respect to PRE-HIIT (IPAQ:3)

When participants performed the aerobic session, cytokines IL-1β, IL-6, IL-17 A (only in IPAQ:3) and IFN-γ decreased in post-exercise condition, meanwhile, cytokines IL-7, IL-8 and TNF-α increased compared to the pre-exercise condition. Nevertheless, differences in the mentioned cytokines were not significant. On the other hand, the anti-inflammatory cytokine IL-2 showed an increase post-exercise compared to the pre-exercise situation, being significant in IPAQ:3 participants. Moreover, IL-4 and IL-10 displayed no significant changes post-exercise. Regarding the effect of aerobic exercise on protein carbonyls, the post-exercise presented no significant changes compared to the pre-exercise situation.

A similar pattern in circulating cytokines compared to the aerobic session was observed in the group when performed the HIIT session (Table 3). Similar to the aerobic exercise, the pro-inflammatory cytokines (IL-1β, IL-17 A, TNF-α and IFN-γ) displayed no significant changes after exercise, compared to the pre-exercise state. Similar observation was noted for IL-6, IL-7 and IL-8. On the other hand, the anti-inflammatory cytokine IL-2 showed a significant increase after exercise, only in IPAQ:3 participants. The rest of anti-inflammatory cytokines (IL-2, IL-4 and IL-10) displayed no significant changes (Table 3). Protein carbonyls decrease post-exercise but differences to pre-exercise situation were not significant.

Finally, with the aim of observing possible baseline changes in the studied cytokines, the group was proposed to undertake a long-term HIIT routine (12 weeks). Only 4 subjects who regularly engaged in intense activity (IPAQ:3) agreed to follow the proposed protocol. In a previous pilot study, we observed that this exercise routine was safe and maintained stable weight and blood glucose levels around 140 md/dL both at the beginning and at the end of the session [24]. Compared to the initial situation (HIIT pre-exercise), only TNF-α presented a significant increase. The rest of pro-inflammatory cytokines (IL-1β, IL-17 A and IFN-γ) displayed no significant changes compared to HIIT pre-exercise situation. IL-2, IL-4, and IL-10 showed non-significant increases compared to the baseline. On the other hand, IL-6, IL-7 and IL-8 are considered as pro-inflammatory, but they can promote other actions, such as muscle repair. For this reason, they were considered as homeostatic in the present report (see Discussion). Except for IL-6, IL-7 and IL-8 presented significant increases after the long-term HIIT routine. Notably, the anti-inflammatory IL-22 was not detected at the baseline or after short-term exercise (aerobic and HIIT). However, after the long-term HIIT protocol, IL-22 could be measured. Finally, protein carbonyls decrease, but not significantly. However, these results should be interpreted with caution, as the number of participants who followed this last part of the intervention was very small. To confirm these findings, additional determinations will be made by increasing the number of participants.

## Discussion

Many studies indicate that exercise can modulate inflammation levels and oxidative imbalance in individuals who regularly engage in physical activity [30, 31]. Under normal circumstances, these changes depend on the type, intensity, and duration of the exercise [32]. The inflammatory damage that occurs after exercise triggers mechanisms for muscle tissue repair to allow post-exercise recovery [32]. Similarly, the free radicals generated after exercise mediate the expression of genes that encode antioxidant proteins [33]. However, when individuals have T1DM, recovery must address additional variables inherent to the disease. Thus, the cytokines present in circulation respond to a wide variety of environmental and internal stressors [34]. All this would explain why the cytokines measured in the subjects participating in this study do not follow a normal distribution. Protein carbonyls were selected as oxidative stress markers because hyperglycemia promotes protein glycation, activating subsequent oxidation [35]. This is a usual event in T1DM [36].

In this context, the cytokines present in the pre-exercise situation follow a slightly different pattern, even though it is the same group of subjects. A 72-h rest period was allowed between the two sessions, during which the subjects were required to rest. However, it should be considered that many molecular parameters follow highly variable patterns during the progression of T1DM, with cytokine profiles being one of them [37]. This could explain why the cytokine patterns in each pre-exercise situation (aerobic vs. HIIT) are not exactly the same. However, each specific post-exercise situation will be compared with its corresponding pre-exercise situation, as the pre-exercise pattern conditions the patterns observed in post-exercise.

The aim of this study was to obtain information on the profiles of circulating cytokines post-exercise in diabetic participants after a HIIT session. A pilot study from our group indicated that HIIT maintained blood glucose levels within safe ranges in these patients [24]. Therefore, it could be an alternative type of exercise for those diabetic individuals who are not attracted to aerobic exercises. However, our initial study could not gather data regarding the patterns of circulating cytokines and oxidative imbalance. Thus, the present study aims to provide information in this regard. To achieve this, subjects who regularly engaged in physical activity were selected, distinguishing between those who exercised moderately (IPAQ:2) and those who exercised intensely (IPAQ:3). This is an important aspect, as intensity can influence the circulating patterns of pro- and anti-inflammatory cytokines [32].

The pro-inflammatory cytokines IL-1β, IL-17 A, IFN-γ, and TNF-α play an instrumental role in the development of T1DM. IL-1β, together with TNF-α, promotes inflammation and the destruction of pancreatic β-cells through apoptotic processes [38]. Additionally, they promote the production of other cytokines, aggravating the inflammatory process. IL-17 A, produced by Th17 cells, also contributes to inflammatory damage, exacerbating the autoimmune process [39, 40]. IFN-γ, produced mainly by cytotoxic T cells and Th1 cells, plays a role in activating macrophages and amplifying the inflammatory response, participating in the direct destruction of β-cells [38]. Therefore, these cytokines create an inflammatory environment in the pancreas that facilitates the autoimmune attack and destruction of insulin-producing cells, leading to the development of T1DM. In the present report, these cytokines exhibit different patterns depending on the type of exercise when comparing pre- and post-exercise situations. However, it should be noted that the observed changes are not significant. Only IFN-γ decreases after aerobic and HIIT exercise no detected for moderate intensity (IPAQ:2), and not significantly for high intensity (IPAQ:3). Therefore, the results suggest that the type of exercise performed appears to maintain a certain stability in these pro-inflammatory cytokines, which seem to increase in diabetics with a more sedentary lifestyle [41]. Exceptionally, IFN-γ is the only inflammatory cytokine that decreases in the post-exercise situation, delaying likely the inflammatory response.

Regarding anti-inflammatory cytokines, IL-2, IL-4, and IL-10 reduce the activity of aggressive Th1 immune cells, promoting tolerance [42] and favoring the proliferation of Th2 cells, particularly IL-4 [43]. IL-4 and IL-10 do not show significant changes after either type of exercise. However, IL-2 shows significant changes after both types of exercise, particularly in subjects who regularly engage in intense physical activity (IPAQ:3). This cytokine promotes the function of regulatory T cells (Tregs), helping to maintain immune tolerance by favoring Th2 levels [42]. In this context, IL-2 appears to play a key role in controlling autoimmunity, thereby contributing to the maintenance of β-cells. Animal models of diabetes have detected antibodies against IL-2, and similar results seem to be reproduced in diabetic patients [44]. Additionally, various studies in non-diabetic active populations indicate that IL-2 is released after exercise, at different times and with varying responses [45]. Higher IL-2 secretions are observed in sedentary individuals following a training program of several weeks. This seems to suggest that these different IL-2 secretion patterns post-exercise depend on the intensity of the activity performed, as well as the regularity with which subjects exercise. The diabetic participants in the present study showed significantly elevated IL-2 levels, particularly in the most active group (IPAQ:3). According to our results, it seems that HIIT could be a good training option for type 1 diabetics, as the post-exercise increase in IL-2 may have an effect on modulating the inflammatory response, which could be key for future therapies against type 1 diabetes.

On the other hand, other cytokines have been determined that, depending on the context in which they act, can exert pro- or anti-inflammatory effects. This is the case for IL-6, IL-7, and IL-8, particularly in the long-term HIIT. For instance, when IL-6 is secreted by T cells and macrophages, it promotes inflammatory processes [46]. However, when this cytokine is produced by muscle, particularly after exercise, it exerts anti-inflammatory effects, counteracting the actions of TNF-α and IL-1β, and activating IL-1ra and IL-10 [10]. However, the half-life of muscle secreted IL-6 is very short (2 min), explaining the no significant changes observed for this particular cytokine in post-exercise condition [47]. IL-7 increases the cytotoxicity of T lymphocytes, stimulates the production of inflammatory cytokines such as IL-1β and TNF-α, and antagonizes IL-4 [48]. On the other hand, IL-7 cooperates with IL-2 and IL-6 in anti-inflammatory processes [48]. Additionally, IL-7 is constitutively produced by stromal cells of the bone marrow, promoting the proliferation and differentiation of future circulating cells [49]. This cytokine only shows significant changes after the long-term HIIT routine in subjects who regularly engage in intense physical activity (IPAQ:3). However, these results should be interpreted with caution, because IL-7 seems to play a key role in the development of T1DM. In this line, the blockade of IL-7 receptor (IL-7Rα) with monoclonal antibodies prevents TIDM development [50]. In order to modulate properly the intensity of exercise, future research is necessary to address this aspect in T1DM subjects. IL-8, on the other hand, is produced by T lymphocytes, alveolar macrophages, neutrophils, endothelial cells, and keratinocytes, among others [51]. In this intervention, IL-8 only increases significantly in the LT-HIIT group. No significant changes appear in the short-term after the AERO or HIIT session. Although it is a pro-inflammatory cytokine, IL-8 promotes angiogenesis in poorly vascularized tissues, such as certain areas of skeletal muscle, which aids in its repair and recovery after exercise [52]. However, a recent study [53] investigates how physical activity and body fat levels affect pro-inflammatory cytokine levels in adults with T1DM. The results suggest that moderate physical activity may reduce low-grade systemic inflammation in these patients, thereby lowering the risk of cardiovascular diseases. Specifically, higher IL-8 levels were observed in inactive men, regardless of adiposity, and in inactive women with normal body fat levels. Based on the study’s findings, moderate-intensity physical activity would likely be the most recommended for individuals with T1DM. In the present report, this is the case of individuals performing exercise at moderate intensity (IPAQ:2). However, IL-8 levels may increase if glycemic control is not properly managed. This was not the case for the subjects participating in our study, who maintained good control of this parameter, which is essential for benefits in physical activity. Nevertheless, some points should be considered. First, the pro-inflammatory action of IL-8 could be counteracted by the increase of IL-22 (see below). Second, the n is very low and studies with significant cohorts are necessary. Third, intervallic moderate routines could be designed for people that is not motivated by aerobic routines and prefer alternative exercise routines. In summary, more research is necessary in this new scientific scenario.

Finally, the most interesting finding is a significant increase in the anti-inflammatory cytokine IL-22, which has been associated with a regenerative role in β-cell [54]. We can hypothesize that programmed exercise generates a circulating environment that could favor preservation of β-cells. In this line, different exercise modalities, such as moderate-intensity continuous training (MICT) and HIIT, modulate circulating IL-22 levels in adults with metabolic syndrome [55]. The findings suggest that both exercises can positively modulate IL-22 levels, contributing to improved metabolic and cardiovascular health. In T1DM context (present report), this is an interesting result. Nevertheless, certain points should be considered. HIIT can offer similar or even superior benefits to MICT in less time, inducing improvements in cardiovascular health. In addition, the anti-inflammatory action of IL-22 could counteract the pro-inflammatory action of TNF-α, IL-7 and IL-8, mentioned before. However, HIIT can be challenging for beginners, with a high risk of injuries. For this reason, HIIT routines require careful supervision. Since it appears that the long-term HIIT routine favored the basal expression of IL-22, this finding highlights the need for future research to elucidate the precise role of this cytokine in T1DM recovery. In addition, these subjects practicing HIIT underwent stable glycemia as well as HbA1c, confirming previous observations [24]. However, the long-term results have been obtained from only in 4 subjects and should be interpreted with caution. Future interventions with a larger number of subjects are needed to address these questions and design interval routines at moderate intensity, that display an anti-inflammatory profile.

## Data Availability

No datasets were generated or analysed during the current study.
